# Development, Characterization, and Validation of an MS2 Phage-Based Armored RNA Control for Schmallenberg Virus Detection

**DOI:** 10.3390/pathogens15080865

**Published:** 2026-08-19

**Authors:** Mengqi Zhao, Yibo Wang, Yifei Xie, Jianshuai Gao, Boyuan Zhang, Huitong Li, Dan Liu, Hui Jiang, Guangzhi Zhang, Pengtao Jiao, Jiabo Ding, Jinling Liu, Qingchun Shen

**Affiliations:** 1 Key Laboratory of Animal Biosafety Risk Prevention and Control (North), Ministry of Agriculture and Rural Affairs, Institute of Animal Sciences, Chinese Academy of Agricultural Sciences, 2 Yuanmingyuan West Road, Beijing 100193, China; zhaomengqicau@163.com (M.Z.); w964461026@163.com (Y.W.); 13107204386@163.com (Y.X.); gaojianshuai2023@163.com (J.G.); zhangboyuan@caas.cn (B.Z.); lht13775652114@163.com (H.L.); 15903971411@163.com (D.L.); jianghui01@caas.cn (H.J.); zgz_126com@126.com (G.Z.); jiaopengtao@caas.cn (P.J.); dingjiabo@caas.cn (J.D.); 2 Key Laboratory of Livestock Infectious Diseases, Ministry of Education, and Key Laboratory of Ruminant Infectious Disease Prevention and Control (East), Ministry of Agriculture and Rural Affairs, College of Animal Science and Veterinary Medicine, Shenyang Agricultural University, 120 Dongling Road, Shenyang 110866, China

**Keywords:** Schmallenberg virus (SBV), MS2 bacteriophage, armored RNA, full-process quality control, matrix tolerance, biosafety

## Abstract

Schmallenberg virus (SBV) is a transboundary animal pathogen that causes reproductive disorders in ruminants, necessitating standardized molecular surveillance. Adhering to the Chinese national standard GB/T 43159—2023, we developed armored RNA quality control materials using MS2 bacteriophage technology targeting the conserved SBV S segment and comprehensively characterized their physicochemical properties. Transmission electron microscopy (TEM) showed icosahedral symmetric virus-like particles (VLPs, approximately 25 nm), with the stock concentration determined to be 3.48 × 10^10^ copies/mL via digital PCR (dPCR). The armored RNA control effectively withstood RNase A degradation and remained stable at 37 °C for over 30 days. In bovine serum matrix simulation assays at identical medium concentrations, the serum armored RNA group (Ct 26.57) was detected 4.89 cycles earlier than the degraded serum naked RNA group (Ct 31.46). This confirms the physical protective efficacy of the MS2 capsid. Concurrently, the serum armored RNA group showed a delay of only 2.17 cycles compared to the aqueous armored RNA group (Ct 24.40), exhibiting a typical matrix effect. Overall, this armored RNA enables full-process quality control encompassing extraction, reverse transcription, and amplification, providing a safe and stable technical reference to support molecular surveillance and diagnostic preparedness for cross-border SBV.

## 1. Introduction

Schmallenberg virus (SBV) belongs to the order *Bunyavirales*, family *Peribunyaviridae*, and is a single-stranded, negative-sense, segmented RNA virus transmitted by insect vectors such as *Culicoides* biting midges [1]. First discovered and named in Germany in 2011, the virus subsequently spread rapidly across Europe, causing severe fever, reduced milk production, and devastating reproductive outcomes, including fetal malformations, stillbirths, and abortions in domestic ruminants, which led to immense economic losses for the European livestock industry [2,3]. Notably, SBV experienced a significant re-emergence and outbreak in Scotland, UK, between 2024 and 2025, demonstrating its active transmission capability and persistent threat [4]. Long-term surveillance data spanning a decade in Sardinia, Italy, indicated that SBV has established a continuous endemic status in the region, with seroprevalence consistently fluctuating between 16% and 22% [5]. The SBV genome consists of three segments: Large (L), Medium (M), and Small (S) [6]. Due to its segmented nature, the virus is highly prone to genetic reassortment and variation, complicating prevention and control strategies [7]. With the increasing frequency of global livestock and poultry trade, SBV has exhibited a distinct potential for transcontinental dissemination [1]. Although SBV has not yet been detected in China, it is categorized as a typical transboundary animal disease (TAD) that poses a critical challenge to epidemiological monitoring and diagnostic preparedness [8]. To counter its persistent prevalence, international research focusing on novel antiviral strategies against SBV has been actively expanding in recent years [9].

Accurate molecular detection represents a pivotal cornerstone for the surveillance and containment of SBV. The Chinese national standard GB/T 43159—2023 has recommended the detection of the highly conserved S segment as the definitive diagnostic criterion [10]. However, as a single-stranded RNA virus, SBV is extremely susceptible to nuclease-mediated degradation during sample collection and transport, frequently yielding false-negative results [11,12]. Current quality control methodologies fail to simultaneously balance biosafety and efficacy: traditional inactivated viruses carry inherent biosecurity risks and batch-to-batch variations [13]; in vitro transcribed RNA (IVT-RNA) exhibits substandard stability and cannot monitor the upstream nucleic acid extraction phase [14]; plasmid DNA lacks validation for the reverse transcription step and is highly prone to causing aerosol contamination [15]. To address these bottlenecks, a “full-process” quality control material is urgently needed. Such a material should possess high environmental stability and robust biosafety, while effectively mimicking the extraction characteristics of wild-type viruses to enhance the reliability of SBV molecular diagnostics.

Armored RNA technology, based on the MS2 bacteriophage platform, provides an ideal paradigm to resolve the aforementioned predicaments [16]. This technology relies on the specific recognition among the coat protein (CP), maturation protein (A protein), and the *pac* site, driving the self-assembly of virus-like particles (VLPs) within *Escherichia coli* and encapsulating the foreign target RNA inside the capsid shell. This unique VLP structure establishes a rigid physical barrier for the internal RNA, exhibiting physical robustness highly analogous to that of native viruses [17]. This platform has been successfully applied to the development of quality controls for various pathogens, including multiple common enteric RNA viruses and severe acute respiratory syndrome coronavirus 2 (SARS-CoV-2), demonstrating excellent versatility and reliability [18,19].

In this study, we developed an armored RNA quality control for full-process SBV detection, adhering strictly to the conserved S-segment target in GB/T 43159—2023. We systematically characterized its morphology, copy number concentration, and thermal stability. Furthermore, its full-process quality control capability—encompassing extraction, reverse transcription, and amplification—was validated using a bovine serum matrix model. The results demonstrate that the developed quality control exhibits robust environmental tolerance and nuclease resistance, thereby providing a safe, stable, and reliable physical reference for emergency epidemic prevention of orthobunyaviruses and early warning of cross-border imported animal diseases.

## 2. Materials and Methods

### 2.1. Target Sequence Design and Primer Synthesis

Based on the genomic sequence of SBV retrieved from GenBank (GenBank accession number KT795146.1), a target fragment (446 bp in length) was designed within the conserved S segment stipulated in the Chinese National Standard (GB/T 43159—2023) [10]. This target gene fragment was chemically synthesized by Sangon Biotech Co., Ltd. (Shanghai, China). The corresponding diagnostic primers (SBV-S-382F and SBV-S-469R) and TaqMan probe (SBV-S-408FAM) strictly conformed to the requirements of GB/T 43159—2023 and were synthesized by Tsingke Biotechnology Co., Ltd. (Beijing, China). The detailed sequence information for these primers and probes is provided in Table 1.

### 2.2. Main Reagents and Instruments

The primary reagents and consumables utilized in this study included PrimeSTAR^®^ Max DNA Polymerase (TaKaRa, Dalian, China; Cat. No. R045); ClonExpress Ultra One Step Cloning Kit (Vazyme, Nanjing, China; Cat. No. C115); HiScript IV All-in-One Ultra RT SuperMix for qPCR (Vazyme, Nanjing, China; Cat. No. R433-01); AIPzol Chloroform-Free Total RNA Extraction Reagent (I-Presci Scientific, Beijing, China; Cat. No. RE205-02); Hieff Unicon^®^ qPCR TaqMan Probe Master Mix (Yeasen, Shanghai, China; Cat. No. 11205ES60); BeyoZonase™ Super Nuclease (Beyotime, Shanghai, China; Cat. No. D7121-25KU); 2× dPCR Cy5.5 Probe Master Mix (Sniper, Suzhou, China; Cat. No. RT017096A); Isopropyl-β-D-thiogalactopyranoside (IPTG; TransGen Biotech, Beijing, China; Cat. No. S31024); and Ribonuclease A (RNase A; TransGen Biotech, Beijing, China; Cat. No. S20227-V2).

The primary instruments and equipment included Applied Biosystems™ 7500 Real-Time PCR System (Thermo Fisher Scientific, Waltham, MA, USA; Model No. 4351106); NanoDrop™ One/OneC Microvolume UV–Vis Spectrophotometer (Thermo Fisher Scientific, Wilmington, DE, USA; Model No. ND-ONEC-W); High-Pressure Microfluidics Nano-Homogenizer (Doning Biotech Co., Ltd., Suzhou, China; Model No. DN-200); HT7700 Transmission Electron Microscope (Hitachi High-Tech, Tokyo, Japan; Model No. HT7700); and Sniper DQ24 Digital PCR System (Suzhou Sniper Medical Technology Co., Ltd., Suzhou, China; Model No. DQ24).

### 2.3. Construction of the Recombinant Expression Vector pET-CPA-SBV

The recombinant plasmid was constructed leveraging the MS2 bacteriophage armored RNA expression system. A 369 bp native target sequence derived from the conserved S segment of SBV (GenBank accession No. KT795146.1, nt 192–560) was amplified using specific cloning primers (N-F and N-R, listed in Table 1) carrying 16 bp and 17 bp terminal vector-overlapping arms (total insert length: 446 bp). The purified PCR product was subsequently cloned into the BamHI and HindIII double-digested and linearized pET-CPA vector via seamless homologous recombination between the MS2 CP and A protein-coding regions. The successful assembly of the recombinant plasmid, designated as pET-CPA-SBV, was verified and confirmed via Sanger sequencing. A detailed schematic plasmid map and the annotated sequence of the insert are provided in Figure S1 and Text S1 of the Supplementary Materials.

### 2.4. Induced Expression and Preparation of Armored RNA Control

The pET-CPA-SBV plasmid was transformed into *Escherichia coli* BL21 (DE3) competent cells. The cells were cultured at 37 °C until the optical density at 600 nm (OD_600_) reached 0.6, followed by the addition of 0.1 mmol/L IPTG to induce expression at 25 °C for 16 h. The bacterial pellets were harvested and disrupted using a high-pressure microfluidics nano-homogenizer. The supernatant was subsequently treated with 30% ammonium sulfate precipitation combined with BeyoZonase™ supernuclease digestion to obtain crude VLP precipitates. The crude sample was resuspended in phosphate-buffered saline (PBS) and further purified via gel filtration chromatography using a Superdex 200 column equilibrated and eluted with a buffer containing 20 mM Tris-HCl and 50 mM NaCl (pH 8.0). Purified armored RNA VLPs were collected and stored in the same buffer at −40 °C for subsequent structural characterizations and performance evaluations. The purity of the CP was evaluated by sodium dodecyl sulfate–polyacrylamide gel electrophoresis (SDS-PAGE).

### 2.5. Morphological Characterization of Armored RNA Particles

The ultrastructure and morphology of the purified armored RNA VLPs were delineated via negative-staining transmission electron microscopy (TEM). The purified sample was dropped onto a charge-neutralized 200-mesh hydrophilic carbon-coated copper grid and allowed to adsorb for 2 min. Subsequently, the specimen was negatively stained with 2% phosphotungstic acid (pH 7.0). The morphology, particle size distribution, and structural integrity of the armored RNA VLPs were observed under a Hitachi HT7700 TEM at an accelerating voltage of 80.0 kV, and representative high-definition micrographs were captured.

### 2.6. Target Sequence Conservancy Analysis

To evaluate target sequence conservancy across SBV isolates and assess potential limitations related to genomic variability, genomic sequences of the SBV S segment deposited in the National Center for Biotechnology Information (NCBI) GenBank database between 1 January 2016, and 8 August 2026, were retrieved (*n* = 146). Multiple sequence alignment was performed using Multiple Alignment using Fast Fourier Transform (MAFFT) (v7.520) and analyzed using SnapGene (v6.0.2) to evaluate sequence identity within the binding regions of the primers and probe specified in GB/T 43159—2023 [10].

### 2.7. Quantitative Analysis and Absolute Copy Number Determination

Total nucleic acids were extracted from the armored RNA preparation using a single extraction reagent (AIPzol reagent) and reverse-transcribed to obtain cDNA. The detectability of the materials was confirmed via the reverse transcription quantitative PCR (RT-qPCR) method using the validated primers and probe set recommended by the national standard GB/T 43159—2023 (Table 1).

Extracted total RNA was reverse-transcribed into cDNA using the HiScript IV All-in-One Ultra RT SuperMix for qPCR. Subsequently, qPCR assays were performed on an Applied Biosystems™ 7500 Real-Time PCR System using the Hieff Unicon^®^ qPCR TaqMan Probe Master Mix. Each 20 μL reaction mixture contained 10 μL of 2× Master Mix, 1.0 μL of forward primer SBV-S-382F (10 μM), 1.0 μL of reverse primer SBV-S-469R (10 μM), 1.0 μL of TaqMan probe SBV-S-408FAM (10 μM), 4.0 μL of cDNA template, and 3.0 μL of nuclease-free water. The thermal cycling conditions were set as follows: preliminary denaturation at 95 °C for 10 min, followed by 45 cycles of 95 °C for 15 s, 64 °C for 20 s, and 72 °C for 30 s, with fluorescence signals collected at 72 °C.

To determine the absolute copy number concentration and evaluate the dynamic range of the digital PCR (dPCR) assay, the stock solutions were subjected to 10-fold serial dilutions. The 10^−4^ dilution (corresponding to a Ct value of approximately 25) was selected for absolute quantification using the Sniper DQ24 Digital PCR system. For dPCR quantification, each 22 μL reaction mixture comprised 11 μL of 2× dPCR Probe Master Mix Plus (Cy5.5) (Sniper, Cat. No. RT017096A), 1.0 μL of forward primer SBV-S-382F (10 μM), 1.0 μL of reverse primer SBV-S-469R (10 μM), 0.5 μL of TaqMan probe SBV-S-408FAM (10 μM), 4.0 μL of cDNA template (from the 10^−4^ dilution), and 4.5 μL of RNase-free water. The thermal cycling conditions were set as follows: droplet stabilization at 60 °C for 5 min; enzyme activation at 95 °C for 5 min, followed by 45 cycles of 95 °C for 10 s and 60 °C for 30 s (with fluorescence signals collected in the Cy5.5 channel).

The RT-qPCR assays across all serial dilutions and the dPCR quantification for the 10^−4^ dilution were both performed with three independent biological replicates, with each sample analyzed in technical triplicate to ensure high precision and reproducibility. Because dPCR was performed on cDNA generated after extraction and reverse transcription, only target RNA molecules that underwent successful extraction and reverse transcription served as templates; thus, the quantified copy number directly reflects the physical target abundance in the evaluated cDNA preparation. According to national standard GB/T 43159—2023, samples with Ct < 38 accompanying specific amplification curves were defined as positive, and the analytical sensitivity (limit of detection, LoD) was defined as the lowest concentration yielding positive detection. The analytical specificity of the standardized primer and probe set was validated under the GB/T 43159—2023 framework.

### 2.8. Nuclease Resistance Assay

To obtain a naked RNA control with the identical sequence, length, and concentration as the internal target of the armored RNA, total RNA lacking capsid protection was extracted directly from the armored RNA using the AIPzol reagent. The armored RNA control and the extracted naked RNA control were independently incubated in PBS (pH 7.4) containing 0.1 µg/mL RNase A at 37 °C for 2 h. Immediately following incubation, AIPzol lysis buffer was added to inactivate RNase A and terminate the reaction through denaturant-mediated protein denaturation. Total nucleic acids were subsequently extracted and analyzed by RT-qPCR in accordance with national standard GB/T 43159—2023 [10]. This assay was performed with three independent biological replicates, and each extracted RNA template was evaluated in technical triplicate by RT-qPCR.

### 2.9. Thermal Stability Evaluation Under Multiple Temperature Regimes

To evaluate the environmental tolerance of the armored RNA as a quality control material under simulated field storage and cold-chain transport scenarios, a long-term storage stability challenge was performed. Aliquots of the armored RNA controls were stored under three distinct temperature conditions: −40 °C, 4 °C, and 37 °C. Samples were harvested on days 10, 20, and 30, with three independent biological replicates established for each time point and temperature condition. Following total nucleic acid extraction and reverse transcription, RT-qPCR was performed to monitor the fluctuations in Ct values based on the national standard GB/T 43159—2023 [10], with each sample assayed in technical triplicate.

### 2.10. Clinical Simulation and Full-Process Performance Validation in Bovine Serum Matrix

To evaluate the matrix resistance and full-process quality control efficacy of the developed material within a complex clinical background, a simulation model was established utilizing a negative bovine serum matrix. Five distinct experimental groups were evaluated: (1) serum armored RNA groups: high, medium, and low concentration working solutions (20 μL each) of the armored RNA control were spiked into undiluted negative bovine serum (180 μL) to achieve final copy concentrations of 3.48 × 10^9^ copies/mL, 3.48 × 10^7^ copies/mL, and 3.48 × 10^5^ copies/mL, designated as Armored RNA (High), Armored RNA (Mid), and Armored RNA (Low), respectively; (2) aqueous armored RNA control group (AR in water (Mid)), in which the identical medium concentration (3.48 × 10^7^ copies/mL) of armored RNA was spiked into nuclease-free water to serve as a matrix-interference-free control; (3) serum naked RNA control group (Naked RNA (Mid)), in which homologous free target RNA was spiked into the negative bovine serum at the same final concentration (3.48 × 10^7^ copies/mL) to serve as a nuclease-sensitive control. The free target RNA was obtained by thermal denaturation of the medium-concentration armored RNA at 95 °C for 15 min to thoroughly disrupt the phage capsid; preliminary experiments in aqueous systems verified that this thermal lysis condition induced no significant shift in Ct values (ΔCt < 0.5) compared to the untreated control, confirming that the heat treatment caused no substantial degradation or interference with the amplification efficiency of the target sequence.

All preparation systems were subsequently incubated at 37 °C for 10 min to thoroughly activate endogenous serum nucleases. Total RNA was then extracted using the AIPzol reagent, wherein strong denaturants complete the dissociation of residual capsid proteins to ensure the complete target RNA release. The resulting RNA eluate was utilized as a template for RT-qPCR amplification, strictly adhering to the GB/T 43159—2023 standard [10]. All evaluation treatments were performed with three independent biological replicates, and each sample was amplified in technical triplicate. By deciphering the distribution profiles of the Ct values across these groups, the concentration-gradient effect, complex matrix tolerance, and physical shielding efficacy of the capsid barrier were systematically characterized.

### 2.11. Data Analysis and Statistics

All experimental assays were performed with three independent biological replicates (*n* = 3), and all RT-qPCR reactions were executed in technical triplicate. Quantitative values are expressed as mean ± standard deviation (SD). Data processing, linear regression (R^2^), and graph rendering were performed using (version 9.0, GraphPad Software, San Diego, CA, USA).

## 3. Results

### 3.1. Target Sequence Conservancy Evaluation

Bioinformatic alignment of the 146 SBV S segment sequences demonstrated that the GB/T 43159—2023 primer and probe binding regions are conserved across the analyzed strains. The forward primer region exhibited 100% sequence identity across all 146 sequences (0 mismatches). For the reverse primer region, 143 of the 146 sequences (97.95%) showed complete sequence matching, with 3 sequences containing a single-nucleotide mismatch (none located at the 3′-ends). In the TaqMan probe binding region, 136 of the 146 sequences (93.15%) displayed complete sequence identity, while 10 sequences possessed a single internal mismatch.

### 3.2. Plasmid Construction and Capsid Protein Expression Verification

**Figure 1:**
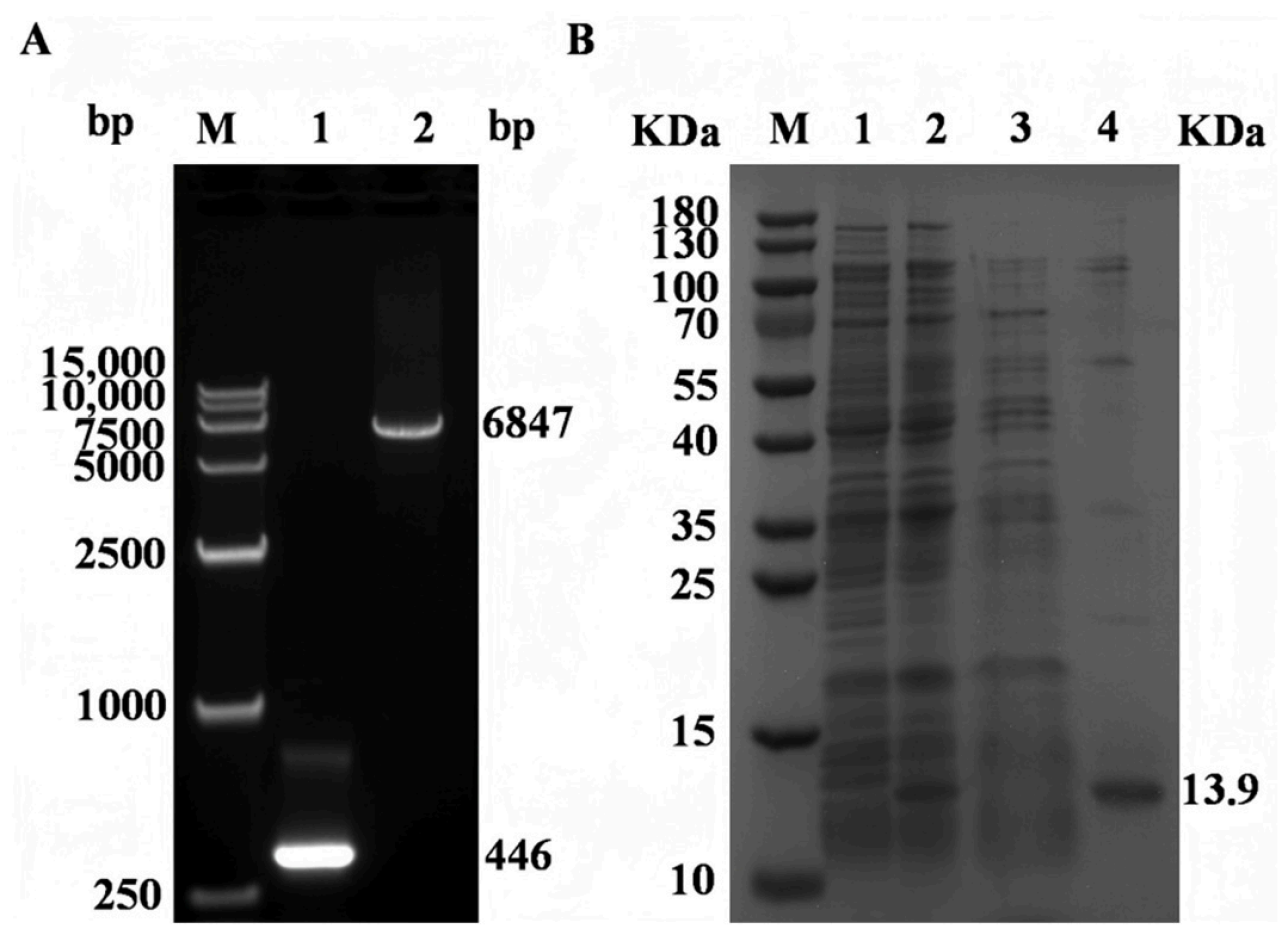
Construction of the recombinant plasmid and verification of capsid protein expression. (**A**) Agarose gel electrophoresis analysis. Lane M: DNA molecular weight marker; Lane 1: PCR product of the Schmallenberg virus (SBV) target sequence (446 bp); Lane 2: PCR product of the pET-CPA vector backbone (6847 bp). (**B**) Sodium dodecyl sulfate–polyacrylamide gel electrophoresis (SDS-PAGE) analysis of the expressed and purified SBV armored RNA. Lane M: protein molecular weight marker; Lane 1: supernatant of the induced empty pET-28a(+) lysate; Lane 2: supernatant of the induced pET-CPA-SBV lysate; Lane 3: pellet of the induced pET-CPA-SBV lysate; Lane 4: purified armored RNA virus-like particles (VLPs) after Superdex 200 gel filtration chromatography.

To verify whether the expression vector was successfully constructed via homologous recombination, PCR amplification was executed using specific primers. The results confirmed the successful assembly of the recombinant expression plasmid pET-CPA-SBV. Agarose gel electrophoresis (Figure 1A) revealed distinct bands at approximately 446 bp and 6847 bp, corresponding to the expected sizes of the SBV insert and the pET-CPA vector backbone, respectively. Sanger sequencing further validated that the SBV gene fragment was correctly inserted into the junction between the CP and A sequences. Subsequent SDS-PAGE analysis demonstrated a specific protein band at approximately 13.9 kDa, consistent with the predicted molecular weight of the MS2 CP, thereby confirming the successful expression of the capsid protein (Figure 1B).

### 3.3. Ultrastructural and Morphological Analysis of Armored RNA Control Particles

**Figure 2:**
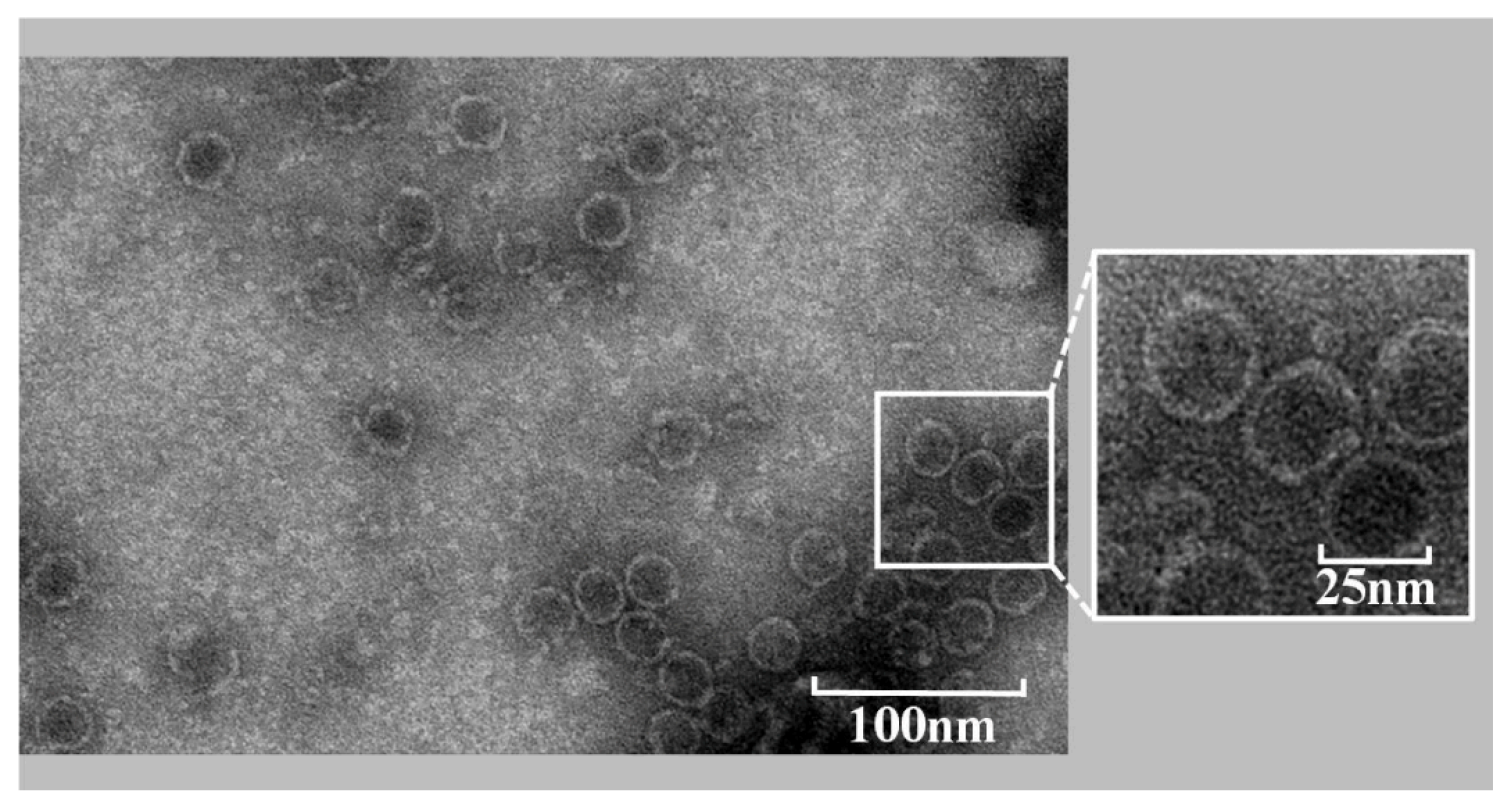
Transmission electron microscopy (TEM) negatively stained micrograph of the SBV armored RNA quality control particles. A representative field of view at high magnification is shown, demonstrating the homogeneous morphology and size (approximately 25 nm) of the self-assembled VLPs. Scale bar: 100 nm.

To observe whether the expressed proteins successfully self-assembled into VLPs and to characterize their morphology, the purified products were examined via TEM. The TEM micrographs (Figure 2) revealed highly homogeneous spherical particles with a diameter of approximately 25 nm, which is consistent with the characteristic size of MS2 bacteriophage virions. These observations confirmed the successful self-assembly of the armored RNA control.

### 3.4. Quantitative Analysis and Absolute Copy Number Determination of Armored RNA Control

**Figure 3:**
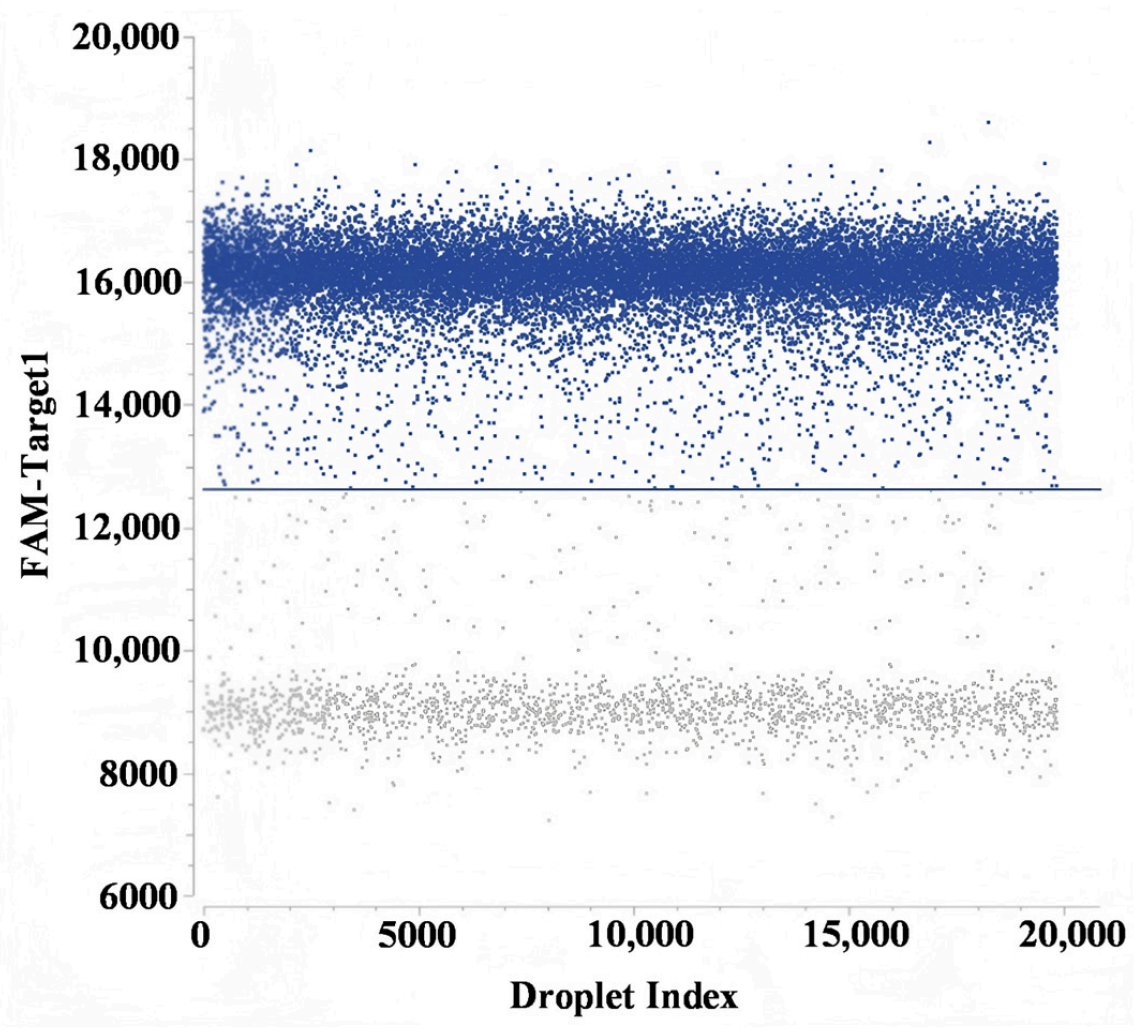
Absolute quantification of the SBV armored RNA quality control material via digital PCR (dPCR). Blue dots indicate positive droplets, grey dots indicate negative droplets, and the blue horizontal line represents the threshold. Data are expressed as mean ± standard deviation (SD, *n* = 3) from three independent biological replicates.

**Figure 4:**
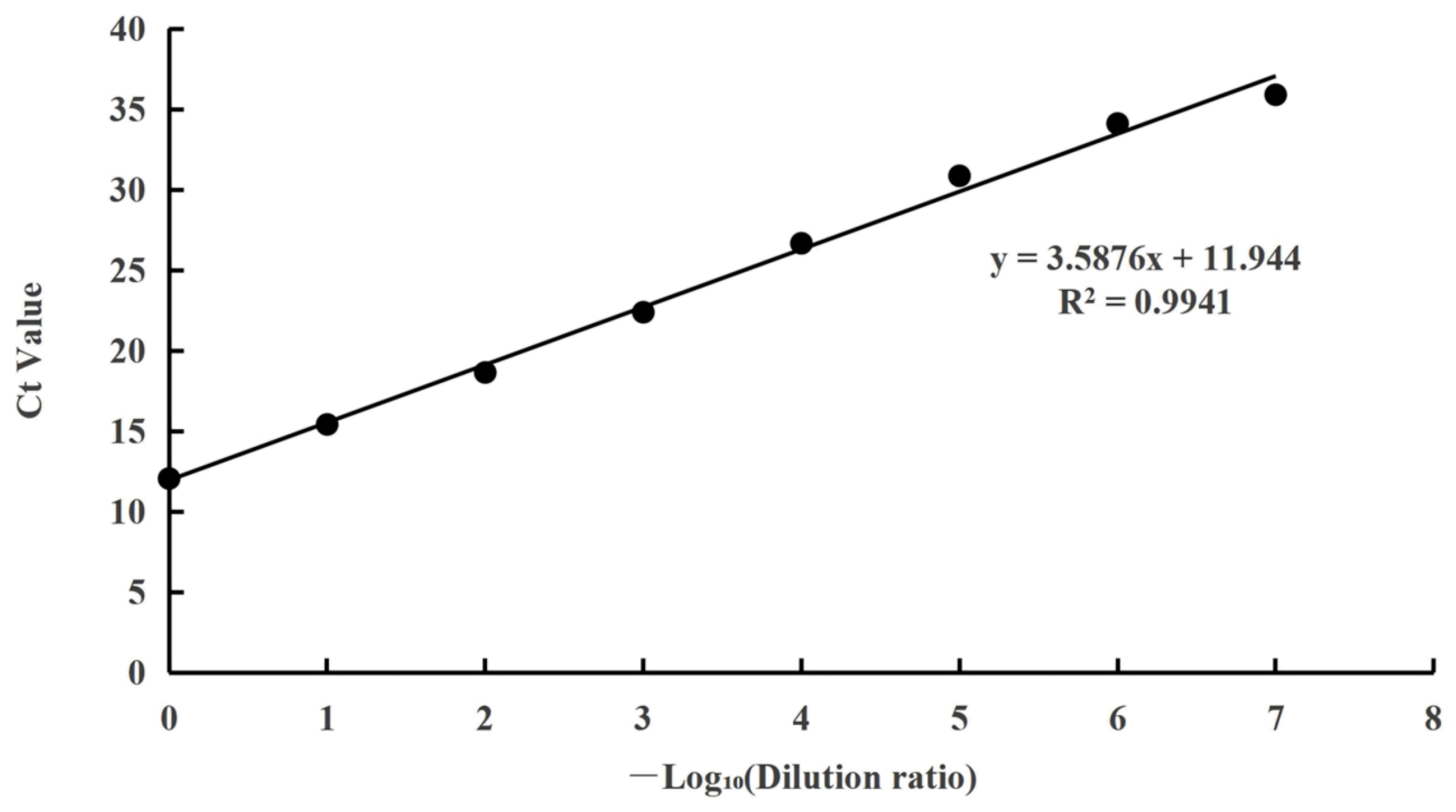
RT-qPCR standard curve of the SBV armored RNA quality control material. The x-axis represents the log_10_ dilution factor, and the y-axis represents the Ct values. The data exhibited a strong linear relationship (R^2^ = 0.9941). Data are representative of three independent biological experiments (*n* = 3), with each dilution analyzed in technical triplicate.

To evaluate the detectability and quantify the copy number concentration of the prepared armored RNA control, RT-qPCR was performed according to national standard GB/T 43159—2023 [10]. Absolute quantification via dPCR determined the stock concentration of the armored RNA control to be 3.48 × 10^10^ copies/mL (Figure 3). In the serial dilution assay, a linear dynamic range was established across the 10^0^ to 10^−7^ dilution, with a coefficient of determination R^2^ = 0.9941 (Figure 4). While linear quantification was maintained up to 10^−7^, positive amplification remained detectable at the 10^−8^ dilution (348 copies/mL, corresponding to 1.39 copies/reaction), yielding mean Ct values between 35.10 and 36.97. Based on the positivity threshold (Ct < 38) specified in GB/T 43159—2023, the analytical sensitivity (LoD) of this standardized assay was confirmed at 348 copies/mL.

### 3.5. Nuclease Resistance Evaluation of the Armored RNA Control

**Figure 5:**
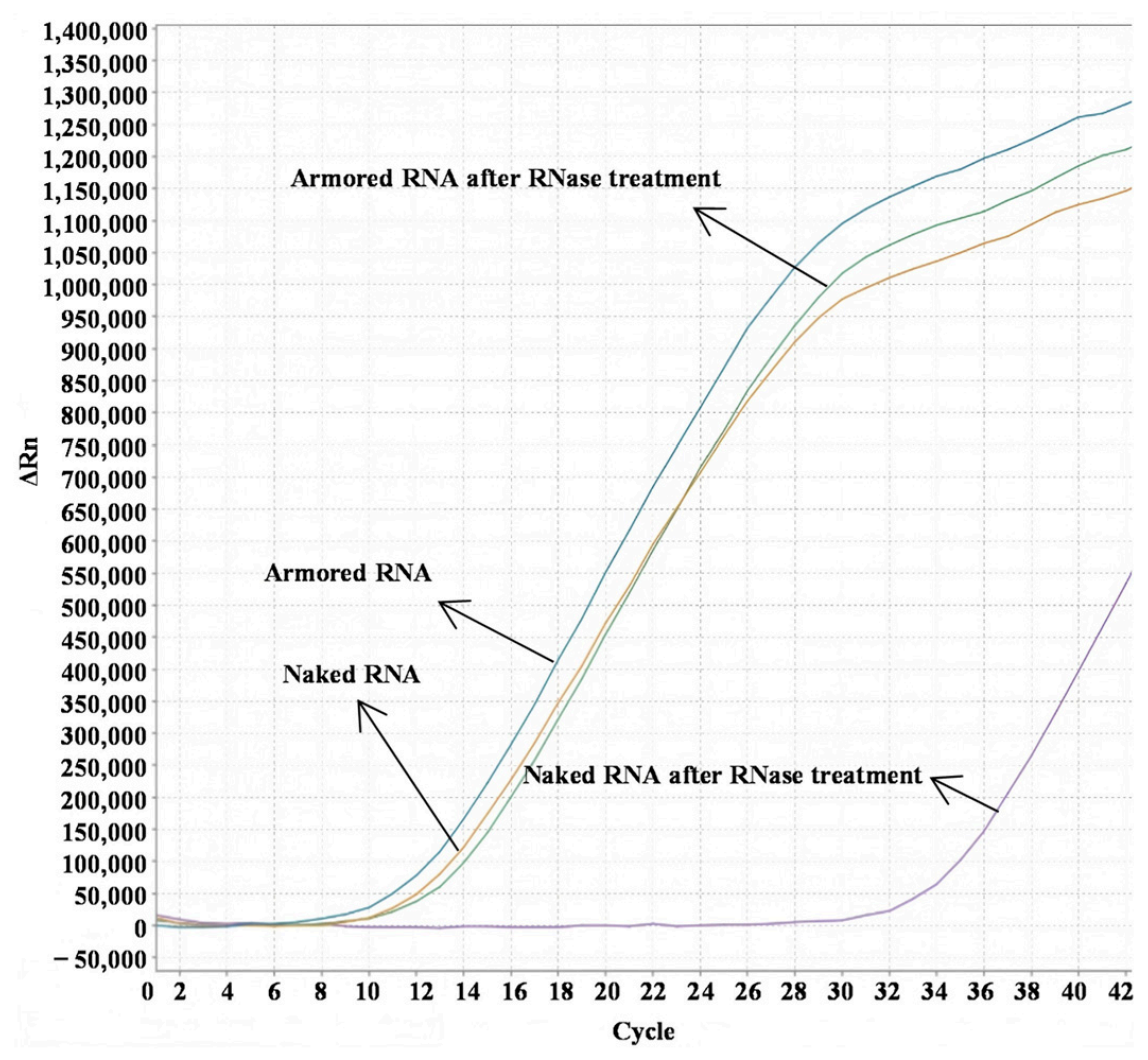
Evaluation of the nuclease resistance of the armored RNA quality control particles against RNase A degradation. The RT-qPCR amplification curves showed that the free RNA control group (Naked RNA) exhibited no amplification signal following RNase A treatment, whereas the armored RNA control group (Armored RNA) yielded almost identical Ct values before and after RNase A treatment. The non-template control (NTC) showed no amplification. Assays were performed in three independent biological replicates (*n* = 3), with each analyzed in technical triplicate.

The RT-qPCR detection revealed that following treatment with a high concentration of commercial RNase A, the target sequences in the free nucleic acid control group (Naked RNA) were completely degraded, with no fluorescence signal detected throughout the amplification cycles (Figure 5). In sharp contrast, the Ct values of the armored RNA control group before and after nuclease digestion were almost superimposed (ΔCt < 1), and their amplification kinetic curves and fluorescence plateaus were congruent. These findings indicate that the assembled MS2 capsid structure provides an effective shield, protecting the internal target nucleic acids against exogenous nuclease-mediated degradation.

### 3.6. Thermal Stability Evaluation of the Armored RNA Control

**Figure 6:**
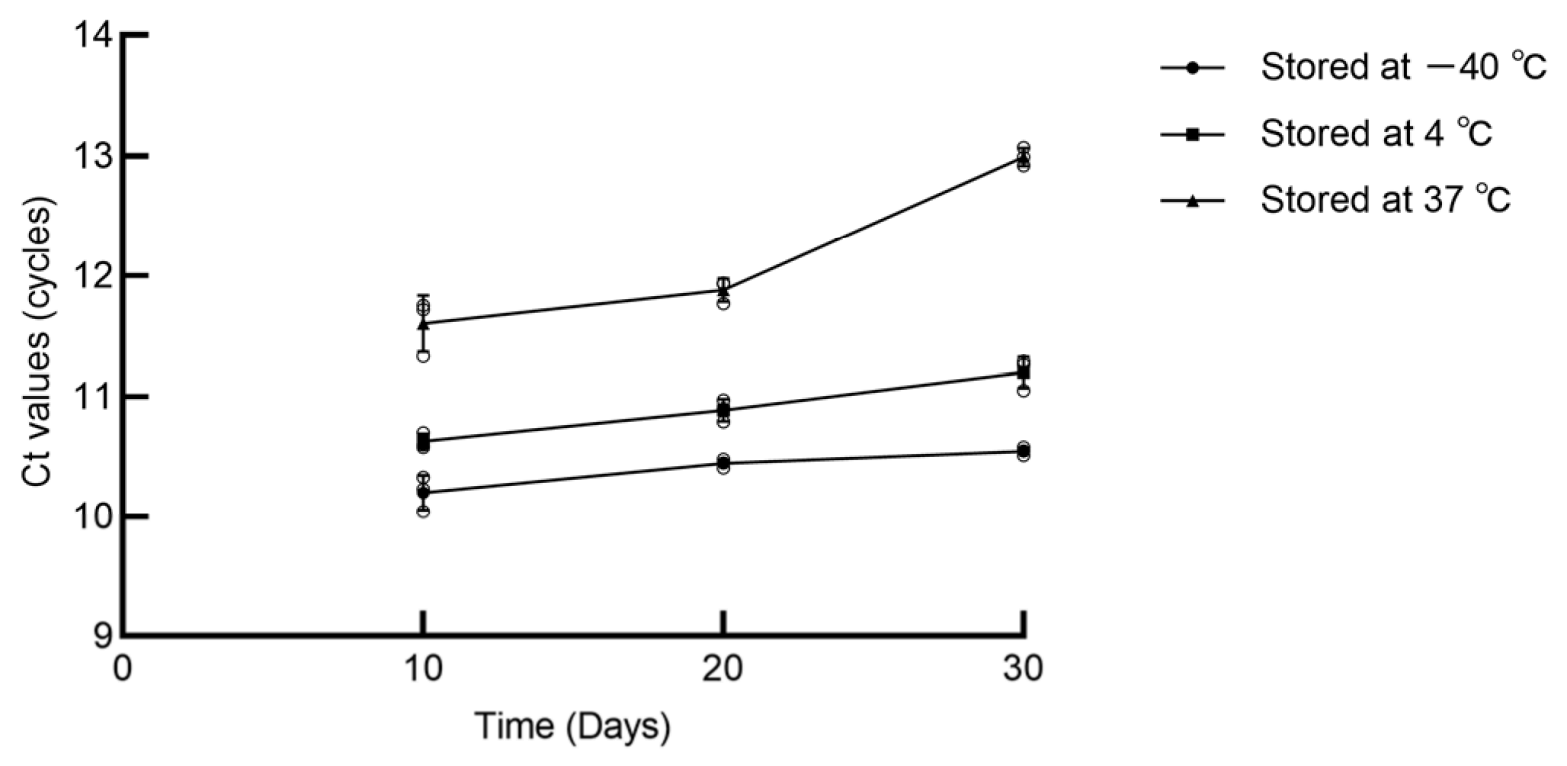
Thermal stability trends of the SBV armored RNA quality control material under multiple temperature conditions. Variation trends of Ct values for the quality control material stored at −40 °C, 4 °C, and 37 °C over a 30-day period are shown. Data are presented as mean ± standard deviation (SD, *n* = 3) from three independent biological replicates, with each sample assayed in technical triplicate; individual data points are shown. No significant deviation in Ct values was observed at all tested temperatures (ΔCt < 3).

Thermal stability monitoring across multiple temperature gradients and an extended timeline revealed that after storage at −40 °C, 4 °C, and 37 °C for 30 days, no significant increase in Ct values was observed across all treatment groups (ΔCt < 3) (Figure 6). Specifically, at Day 30, the mean Ct values (with 95% confidence intervals) were 10.55 (95% CI: 10.46–10.64) for −40 °C, 11.20 (95% CI: 10.88–11.52) for 4 °C, and 12.99 (95% CI: 12.80–13.18) for 37 °C. Under the −40 °C and 4 °C preservation conditions, the stability profiles remained virtually flat. Even after long-term exposure to an elevated temperature of 37 °C, the quality control material maintained its structural integrity and good quantitative reproducibility. Taken together, these data demonstrate that the armored RNA control possesses good tolerance against complex transport and storage environments, highlighting its potential to reduce the reliance of routine molecular surveillance on cold-chain logistics.

### 3.7. Clinical Serum Matrix Simulation and Full-Process Monitoring Capability Evaluation

**Figure 7:**
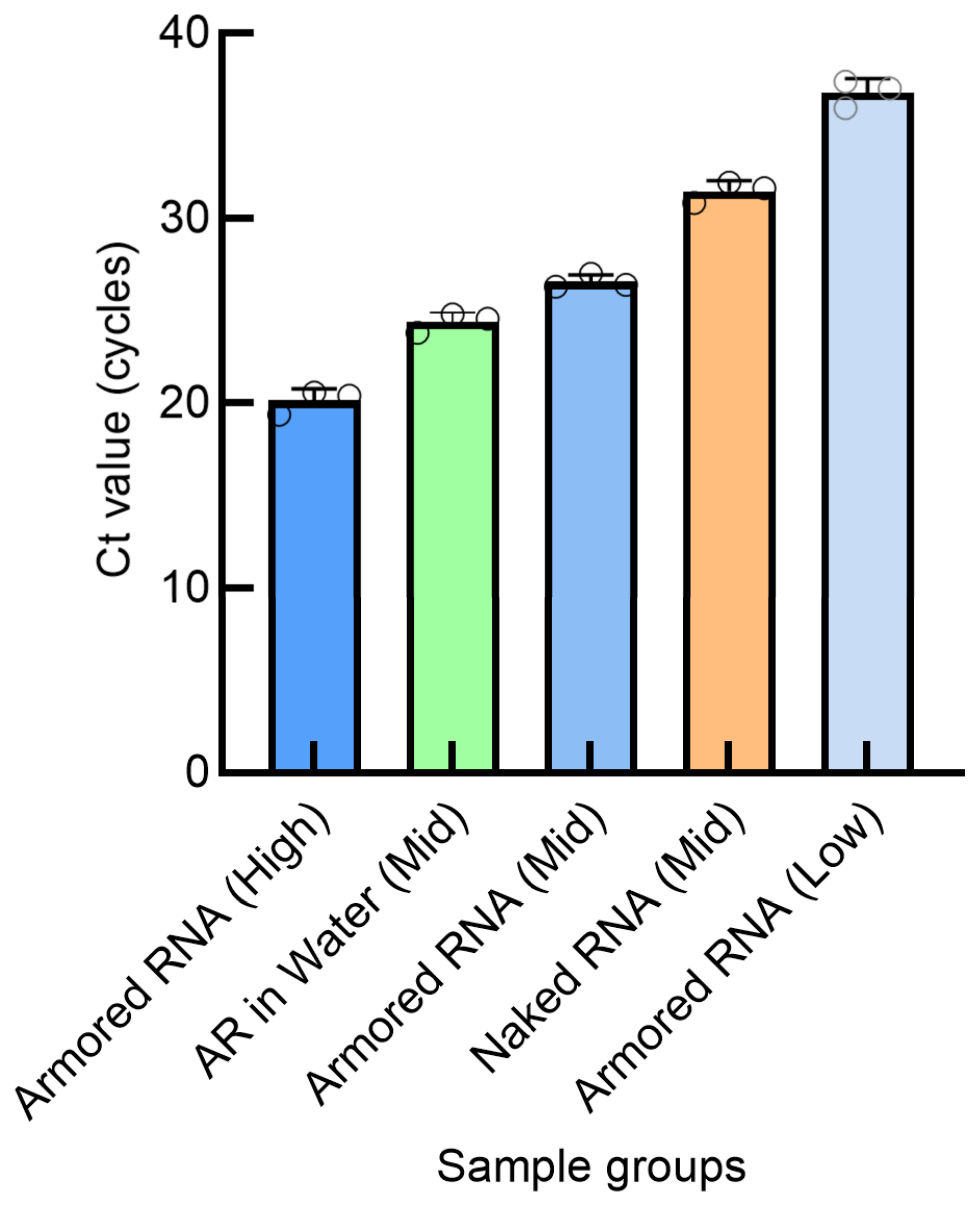
The RT-qPCR detection results of the SBV armored RNA quality control material in a bovine serum matrix. Data are presented as mean ± standard deviation (SD, *n* = 3) from three independent biological replicates, with each sample assayed in technical triplicate; individual data points are shown. The parallel NTC exhibited no amplification signal throughout the cycles.

The RT-qPCR detection results demonstrated that the prepared armored RNA control exhibited a clear concentration response and sample format differences within complex biological matrices (Figure 7). In the armored RNA groups spiked in a bovine serum background, a stepwise decrease in control concentration (from 3.48 × 10^9^ copies/mL to 3.48 × 10^5^ copies/mL) yielded a distinct gradient increase in Ct values across the high, medium, and low concentration groups (Armored RNA (High), Armored RNA (Mid), and Armored RNA (Low)), with mean values of 20.13 (95% CI: 18.55–21.72), 26.57 (95% CI: 25.67–27.48), and 36.79 (95% CI: 34.94–38.65), respectively. This confirms that the armored RNA control maintains a consistent dose–response relationship within the serum matrix. At the identical initial medium concentration level, clear sample format effects were observed across different vectors and environmental conditions. To evaluate the physical protective efficacy of the viral capsid, the armored RNA in serum was compared with the naked RNA control: the serum armored RNA group (Armored RNA (Mid)) yielded a Ct value of 26.57, whereas the serum naked RNA control group (Naked RNA (Mid)), which lacked capsid shielding, exhibited a Ct value of 31.46 (95% CI: 30.03–32.89). This delay of 4.89 cycles (ΔCt = 4.89) reflects the degradation of the thermally released bare RNA by endogenous serum nucleases, indicating the protective role of the MS2 capsid barrier for the encapsulated nucleic acid. To assess the interference of the serum matrix, the serum armored RNA was compared with the armored RNA in pure water: the pure water group (AR in Water (Mid)) yielded a mean Ct value of 24.40 (95% CI: 23.12–25.67), compared to 26.57 for the serum group (Armored RNA (Mid)). This difference of 2.17 cycles (ΔCt = 2.17) points to a degree of inhibition exerted by the serum matrix on the subsequent extraction or amplification steps.

Furthermore, the small standard deviations observed across three independent biological replicates (SD = 0.36 for the mid-concentration group; SD = 0.75 for the low-concentration group) indicate good reproducibility of the assay system under our experimental conditions. Taken together, these findings demonstrate that the armored RNA control successfully mimics the behavior of clinical specimens in a serum matrix, supporting its applicability for full-process quality control encompassing extraction, reverse transcription, and amplification.

## 4. Discussion

SBV, as a typical TAD, poses a continuous threat that highlights the need for molecular surveillance and diagnostic preparedness [8]. Establishing a standardized, full-process molecular detection system is critical for effective early warning and control of the virus. In this study, an SBV armored RNA quality control material was prepared using MS2 bacteriophage armored RNA technology. This technology relies on the artificial construction of non-infectious armored RNA quality control materials (VLPs) that morphologically resemble native bacteriophages while encapsulating exogenous target RNA, combining structural stability, biosafety, and cost-effectiveness in preparation. Compared with enveloped biomimetic platforms such as lentiviral vectors or lipid nanoparticles, the MS2 platform circumvents complex eukaryotic expression systems and enables high-yield preparation under BSL-1 conditions [17,19]. The MS2 capsid proteins form a stable physical barrier protecting the internal RNA cargo [20,21], allowing the armored RNA control prepared in this study to closely mimic the physical properties of native viruses. This control material can be synchronously subjected to clinical sample pre-treatments including lysis, enzymatic digestion, and nucleic acid purification, thereby meeting the requirements for full-process molecular assay quality control. In addition, the sealed icosahedral capsid architecture effectively mitigates the risk of aerosol contamination commonly associated with traditional plasmid DNA controls during routine handling, which contributes to improved biosafety in high-throughput testing at ports and grassroots laboratories.

The core performance of this quality control material was validated using a bovine serum matrix simulation model. At identical medium concentrations, the serum-spiked Naked RNA (Mid) group exhibited a significantly higher Ct value (31.46) compared to the Armored RNA (Mid) group (26.57, ΔCt = 4.89), clearly demonstrating the physical protective efficacy of the MS2 capsid. Meanwhile, the Ct value of the serum-spiked armored RNA group was delayed by 2.17 cycles relative to the pure water control group (24.40). This typical matrix effect [12,22] likely results from steric hindrance caused by high-concentration serum proteins or the co-purification of trace endogenous inhibitors (such as heme or peptides) that interfere with downstream amplification [23]. Notably, the dissociation property of the MS2 capsid within highly denaturing lysis systems [20] rules out the possibility that incomplete capsid rupture contributed to the observed delay. The target nucleic acid remained detectable in the serum naked RNA group after incubation at 37 °C for 10 min (Ct = 31.46), suggesting that the degradation efficiency of endogenous RNases may be constrained by macromolecular steric hindrance rather than being complete. This observation reflects the inherent complexity of RNA virus detection in authentic clinical specimens. The stable amplification of the armored RNA group under matrix interference confirms its utility as a full-process quality control tool for SBV detection and suggests its potential application as a surrogate model for assessing the matrix tolerance of specific extraction and amplification workflows [24]. Furthermore, the Ct values of the control material showed no significant change after storage at 37 °C for 30 days, with thermal stability comparable to that of classical MS2 armored RNA products [20]. This structural stability is governed by non-covalent interactions among capsid protein subunits and the steric hindrance of surface peptide loops [25,26], an advantage that reduces dependence on cold-chain logistics and better accommodates the needs of grassroots and field detection settings. Overall, the engineered armored RNA control demonstrates measurable performance advantages over naked RNA across three key dimensions. In terms of reproducibility, the physical protection provided by the MS2 capsid minimizes quantitative fluctuations caused by potential RNase interference during sample handling, maintaining low variation across biological replicates (SD < 0.5); regarding stability, the armored RNA exhibits minimal Ct shifts before and after high-concentration RNase A treatment (ΔCt < 1) and retains structural stability at 37 °C for over 30 days (ΔCt < 3), mitigating the temperature sensitivity associated with naked RNA; regarding extraction stability, while naked RNA cannot provide monitoring metrics for sample lysis and extraction steps and is susceptible to endogenous nuclease activity in serum matrices (resulting in a Ct delay of 4.89 cycles), the armored RNA undergoes complete capsid dissociation under strong chaotropic lysis conditions to release the target sequence, thereby offering a reliable full-process reference across the extraction, reverse transcription, and amplification stages.

In global surveillance efforts targeting the order *Bunyavirales* and other segmented RNA viruses, balancing the high genetic variability of viral genomes with the structural stability of quality control materials remains a technical challenge in the development of such controls. Currently, detection of viruses within the same order or family—such as Crimean–Congo hemorrhagic fever virus (CCHFV) and Rift Valley fever virus (RVFV)—commonly employs IVT-RNA or double-stranded DNA fragments as positive controls [27]. However, both traditional control types harbor certain limitations: IVT-RNA suffers from poor stability and cannot monitor the nucleic acid extraction step, while DNA controls are inherently unable to validate the reverse transcription stage. Eukaryotic pseudovirus platforms have emerged in recent years but are similarly constrained by complex preparation procedures, relatively high costs, and insufficient thermal stability [28].

The selection of molecular targets and accurate quantification determine the applicability of quality control materials. The SBV genome consists of three segments—L, M, and S—a structure that predisposes the virus to genetic reassortment and evolutionary variation [6,7]. Nevertheless, the nucleoprotein (*N*) gene encoded by the S segment exhibits relatively high sequence conservation throughout its evolution [29]. Following the Chinese national standard GB/T 43159—2023, which adopts the technical framework for RT-qPCR detection established by the World Organisation for Animal Health (WOAH) [10,30], this study selected a conserved region of the S segment as the encapsidation target, thereby reducing the potential interference of minor viral genetic variations with the quality control system. Alignment with these international diagnostic principles supports the technical compatibility and applicability of the control material for cross-regional disease surveillance and inter-laboratory value comparisons. For quantification, dPCR was employed for absolute quantification, a method that does not rely on standard curves. The results showed that the copy number concentration of the stock solution was 3.48 × 10^10^ copies/mL. This high-titer stock solution can support large-scale sample screening while providing a stable quantitative reference for laboratory proficiency testing and external quality assessment. Notably, potential technical differences between physical copy numbers quantified by dPCR and detectable copy numbers assessed by RT-qPCR warrant consideration [31,32]. While dPCR provides absolute quantification based on micro-partition end-point signal counting independent of standard curves, RT-qPCR quantification relies on calibration standards and target amplification efficiency [31]. Discrepancies between these two platforms typically stem from variations in reverse transcription efficiency, nucleic acid recovery losses during extraction, and differences in the spatial accessibility of the target RNA to primers and probes [32]. In a quality control framework, dPCR serves primarily for the initial reference value assignment of physical concentrations, whereas RT-qPCR reflects the functionally amplifiable template load within a specific assay system. Clarifying this technical distinction facilitates a more objective interpretation and application of armored RNA control data across different quantification platforms.

However, certain limitations regarding complex matrix validation, single-platform validation, multi-target coverage, and packaging efficiency should be acknowledged. Given that SBV has not yet been introduced into China and remains an exotic animal disease with no domestic cases, access to authentic clinical positive specimens was limited. Therefore, this study validated the performance of the control material only in a liquid-phase system using a bovine serum spiking model. Although this simulated system confirmed the protective effect of the capsid against nucleases in liquid environments, its physicochemical properties differ from those of solid clinical specimens such as fetal tissues or placental chorionic villi from naturally infected animals. Thus, future international collaborative efforts are needed to obtain authentic tissue samples from endemic areas, enabling further investigation into the nucleic acid release kinetics and full-process recovery of the MS2 capsid within solid tissue lysis systems, as well as quantitative analysis of extraction biases across different matrices to establish correction factors for precise detection in complex clinical samples. In addition, the nucleic acid extraction and quantitative analysis in this initial study were performed using a single extraction reagent (AIPzol reagent) and a single RT-qPCR system (Applied Biosystems 7500 Real-Time PCR System). Although this combination effectively demonstrated capsid disruption, target RNA release, and amplification, future studies will systematically evaluate the performance of this armored RNA control across diverse commercial extraction kits (such as column-based and automated magnetic bead-based platforms) and various RT-qPCR instruments to further confirm its multi-platform compatibility in clinical diagnostic workflows. Furthermore, the packaging efficiency of the armored RNA—defined as the proportion of MS2 VLPs successfully loaded with the target S-segment RNA—was not quantitatively evaluated in this study. Although dPCR provided the absolute concentration of encapsulated target RNA in the stock solution (3.48 × 10^10^ copies/mL), the fraction of total VLPs that were actually loaded remains undetermined. Low packaging efficiency could reduce the yield of loaded VLPs, potentially impacting the cost-effectiveness and scalability of large-scale production [33].

Addressing the risk of diagnostic escape caused by genetic reassortment in segmented bunyaviruses, the development of a multi-target integrated second-generation armored RNA quality control system represents a core direction for our future research. Long heterologous RNA fragments from the M or L segments may alter the spatial conformation of prokaryotic transcripts, which could interfere with *pac* site-mediated capsid self-assembly and potentially exceed the packaging capacity of the bacteriophage capsid. In this context, the successful single S-segment encapsidation and absolute quantification accomplished in this study lay a foundation for subsequent optimization of multi-fragment assembly workflows. Building on the existing technical framework, we plan to employ multi-fragment homologous recombination to integrate the core conserved regions of the SBV M segment and key sequences of the L segment into the pET-CPA vector and to screen for transcript combinations suitable for multi-target co-encapsidation. Theoretically, this control material is expected to adapt to the full-process quality control of mainstream multiplex RT-qPCR detection technologies; however, its practical performance remains to be validated in future experiments, as corresponding tests were not conducted in the present study. Once realized, this platform could help address monitoring blind spots caused by segmented viral variation.

## 5. Conclusions

In summary, this study successfully developed an SBV armored RNA quality control material capable of full-process quality monitoring across the nucleic acid extraction, reverse transcription, and amplification steps. This material can serve as a quantitative reference for standardized SBV molecular surveillance and provides a strategic reference and technical framework for the development of quality control materials for other emerging and re-emerging segmented RNA viruses.

## Figures and Tables

**Table 1:** Primers and probes used for pET-CPA-SBV plasmid construction and RT-qPCR detection.

Primer/Probe Name	Nucleic Acid Sequence (5′ → 3′)	Product Size (bp)
N-F	CATCTACTAAGGATCCCTACATAAGACGGCACAACCAA	446
N-R	ATATCTCCTTCAAGCTTTCAGCAGTCATTGTTCCATAGC	
SBV-S-382F	TCAGATTGTCATGCCCCTTGC	88
SBV-S-469R	TTCGGCCCCAGGTGCAAATC	
SBV-S-408FAM	FAM-TTAAGGGATGCACCTGGGCCGATGGT-BHQ1	

Note: N-F/R: Primers utilized for recombinant vector construction; the underlined sequences indicate the homologous arms for seamless cloning/homologous recombination. FAM:6-carboxyfluorescein (FAM)-labeled. BHQ1: Black Hole Quencher 1. SBV_S series: The standard reverse transcription quantitative PCR (RT-qPCR) primer–probe set targeting the S gene, as recommended by the Chinese National Standard (GB/T 43159—2023).

## Data Availability

The data presented in this study are available within the article.

## Supplementary Materials

The following supporting information can be downloaded at https://www.mdpi.com/article/10.3390/pathogens15080865/s1, Figure S1: Schematic map of the recombinant expression plasmid pET-CPA-SBV; Text S1: Nucleotide sequence of the 446 bp insert containing the SBV S-segment target.

## Abbreviations

The following abbreviations are used in this manuscript:

BSL-1	biosafety level 1
CCHFV	Crimean–Congo hemorrhagic fever virus
CP	coat protein
A protein	maturation protein
PCR	polymerase chain reaction
RT-qPCR	reverse transcription quantitative PCR
dPCR	digital PCR
IVT-RNA	in vitro transcribed RNA
NTC	non-template control
RVFV	Rift Valley fever virus
SARS-CoV-2	severe acute respiratory syndrome coronavirus 2
SBV	Schmallenberg virus
SDS-PAGE	sodium dodecyl sulfate–polyacrylamide gel electrophoresis
TAD	transboundary animal disease
TEM	transmission electron microscopy
VLPs	virus-like particles
NCBI	National Center for Biotechnology Information
IPTG	isopropyl β-D-1-thiogalactopyranoside
MAFFT	multiple alignment using fast Fourier transform
