# Supplementary material for: Development, Characterization, and Validation of an MS2 Phage-Based Armored RNA Control for Schmallenberg Virus Detection
Source: Pathogens. 2026 Aug 19;15(8):865. doi: 10.3390/pathogens15080865 (PMC13516532; doi:10.3390/pathogens15080865)
Supplement: Supplementary file 1 [file pathogens-15-00865-s001.zip › pathogens-4457664-supplementary.pdf]

Development, Characterization, and Validation of an MS2 Phage-Based Armored RNA Control for Schmallenberg Virus Detection

Mengqi Zhao[1,2†], Yibo Wang[1†], Yifei Xie[1], Jianshuai Gao[1], Boyuan Zhang[1], Huitong Li[1], Dan Liu[1], Hui Jiang[1], Guangzhi Zhang[1], Pengtao Jiao[1], Jiabo Ding[1], Jinling Liu[2,*] and Qingchun Shen[1,*]

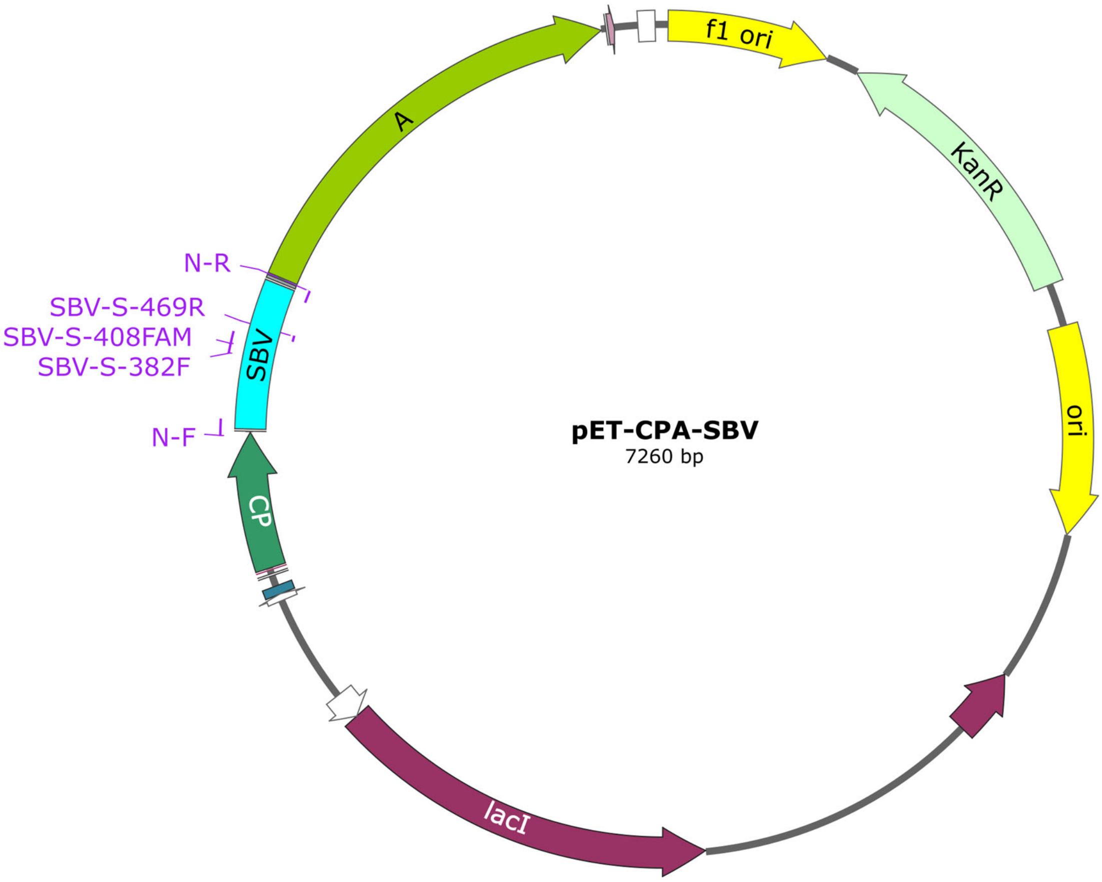

Figure S1. Schematic map of the recombinant expression plasmid pET-CPA-SBV. The 446 bp insert—comprising the 369 bp native Schmallenberg virus (SBV) S-segment target sequence (corresponding to nt 192–560 of GenBank accession No. KT795146.1) and terminal cloning frameworks (containing 16 bp and 17 bp vector-homologous overlap arms)—was seamlessly integrated between the MS2 CP and A protein coding regions within the pET-CPA vector. The relative positions of the seamless cloning primers (N-F and N-R), diagnostic RT-qPCR primers (SBV-S-382F and SBV-S-469R), and TaqMan probe (SBV-S-408FAM) are indicated.

Text S1. Nucleotide sequence of the 446 bp insert containing the SBV S-segment target. The complete nucleotide sequence (5′ →3′) of the synthesized 446 bp insert is provided below, including the 369 bp native SBV S-segment sequence and the terminal cloning flanks:
5'-CATCTACTAAGGATCCCTACATAAGACGGCACAACCAAGTGTCGATCTTACTTTTGGTGGGGT
CAAATTTACAGTGGTTAATAACCATTTTCCCCAATATGTCTCAAGTCCTGTGCCAGACAATGCCA

TTATACTTCACAGGATGTCAGGATATCTAGCACGTTGGATTGCTGATACATGCAAGGCTAGTGT
CCTCAAACTAGCTGAAGCTAGTGC<u>TCAGATTGTCATGCCCCTTGC</u>TGAGG**TTAAGGGATGCAC**
CTGGGCCGATGGTTATACAATGTATCTTG<u>GATTTGCACCTGGGGCCGAA</u>ATGTTCCTTGATGCT
TTTGACTTCTATCCACTAGTTATTGAAATGCATAGGGTCCTCAAGGACAATATGGATGTAAATTT
TATGAAAAAAGTCCTCCGCCAACGCTATGGAACAATGACTGCTGAAAGCTTGAAGGAGATAT–
3'

Note:
The 369 bp core region derives entirely from the native SBV S-segment (GenBank: KT795146.1, nt 192–560). The 88 bp diagnostic RT-qPCR target region recommended by Chinese National Standard GB/T 43159—2023 is indicated within the sequence. The diagnostic forward primer (SBV-S-382F) and reverse primer (SBV-S-469R) binding regions are underlined, and the TaqMan probe (SBV-S-408FAM) binding site is indicated in bold. The terminal sequences at the 5' end (CATCTACTAAGGATCC, 16 bp) and 3' end (AAGCTTGAAGGAGATAT, 17 bp) represent the vector-homologous overlap arms carried by the cloning primers (N-F and N-R) for seamless recombination into the pET-CPA vector.
